# Maternal carbohydrate intake during pregnancy is associated with child peripubertal markers of metabolic health but not adiposity

**DOI:** 10.1017/S1368980021004614

**Published:** 2022-09

**Authors:** Molly C Mulcahy, Martha Maria Tellez-Rojo, Alejandra Cantoral, Maritsa Solano-González, Ana Baylin, Dave Bridges, Karen E Peterson, Wei Perng

**Affiliations:** 1 Department of Nutritional Sciences, University of Michigan School of Public Health, Ann Arbor, MI, USA; 2 Center for Nutrition and Health Research, Instituto de Salud Pública, Cuernavaca, Morelos, Mexico; 3 Department of Health, Universidad Iberoamericana, Prolongación Paseo de Reforma 880, Lomas de Santa Fe, Ciudad de México, C.P. 01219, Mexico; 4 Department of Epidemiology, Colorado School of Public Health, Anschutz Medical Campus, Aurora, CO, USA; 5 Department of Epidemiology, University of Michigan School of Public Health, Ann Arbor, MI, USA; 6 Department of Environmental Health Sciences, University of Michigan School of Public Health, Ann Arbor, MI, USA

**Keywords:** Childhood obesity, Adolescence, Carbohydrate, Nutritional epidemiology, Adiposity, Metabolic health

## Abstract

**Objective:**

To examine the associations of trimester-specific maternal prenatal carbohydrate (CHO) intake with offspring adiposity and metabolic health during peripuberty.

**Design:**

Prospective cohort study in which maternal dietary intake was collected via validated FFQ during each trimester. Offspring adiposity and metabolic biomarkers were evaluated at age 8–14 years. We used multivariable linear regression to examine associations between total energy-adjusted maternal CHO intake and offspring BMI *z*-score, skinfold thickness and metabolic syndrome risk *z*-score calculated as the average of waist circumference, fasting glucose, fasting C-peptide, TAG:HDL and systolic blood pressure + diastolic blood pressure/2.

**Setting:**

Mexico City, Mexico

**Participants:**

237 mother–child pairs in the Early Life Exposure in Mexico to Environmental Toxicants cohort.

**Results:**

We found non-linear associations of maternal CHO intake during pregnancy with offspring metabolic health during peripuberty. After adjusting for maternal age, and child age, sex and pubertal status, children whose mothers were in the fourth *v*. first quartile of total CHO intake during the third trimester had 0·42 (95 % CI –0·01, 0·08) ng/ml lower C-peptide and 0·10 (95 % CI –0·02, 0·22) units lower C-peptide insulin resistance (CP-IR). We found similar magnitude and direction of association with respect to net CHO intake during the first trimester and offspring C-peptide and CP-IR. Maternal CHO intake during pregnancy was not associated with offspring adiposity.

**Conclusions:**

In this study of mother–child pairs in Mexico City, children born to women in the highest quartile of CHO intake during pregnancy had lowest C-peptide and CP-IR during peripuberty. Additional research is warranted to replicate and identify mechanisms.

The incidence of obesity has increased in the last three decades, now pervading paediatric populations, with nearly 24 % of children classified as overweight or obese worldwide in 2013^(1,2)^, which is similar to obesity and overweight prevalence in Mexico^(3)^. In parallel with the trends in childhood obesity, metabolic conditions that have historically been confined to adult populations now afflict children and adolescents. For example, diagnoses of high blood pressure, non-alcoholic fatty liver disease, sleep apnoea, cardiovascular risk and type 2 diabetes have increased among children and adolescents in recent decades^(4,5)^. The younger age of onset of such metabolic conditions not only increases economic and healthcare burden but also has the potential to adversely affect the health of future generations^(6)^.

Maternal nutrition during pregnancy is an important determinant of offspring phenotype and health^(7–9)^. Of particular interest is carbohydrate (CHO) intake given the importance of glycaemic regulation to fetal growth and development^(10,11)^. It is well established that maternal diet during pregnancy can influence metabolic phenotype in the offspring^(7,8)^. Studies evaluating the role of maternal diet during gestation on offspring adiposity and metabolic risk have largely focused on protein and fat consumption, leaving the role of CHO intake largely unstudied^(7,12,13)^. To date, the few investigations relating maternal CHO intake to offspring health have focused on health outcomes during infancy or early childhood. In a study of 320 mother–child pairs in the Growing Up in Singapore Towards health Outcomes cohort, Chen *et al*. examined associations of maternal macronutrient intake at 26–28 weeks’ gestation with offspring peak BMI in infancy^(14)^, an early growth milestone linked to future adiposity and metabolic risk^(15)^. The researchers found that higher ratio of CHO-to-protein intake during pregnancy was related to greater magnitude of peak BMI during infancy – an association that was driven primarily by sugar intake^(14)^. Some studies have also used the glycaemic index as a proxy for the physiological effects of CHO intake. For example, Scholl *et al*. found that greater consumption of low glycaemic index foods during pregnancy was correlated with greater prevalence of small for gestational age infants in a prospective study of 1802 mother–infant pairs in Camden, New Jersey^(16)^. Taken together, these studies indicate the relevance of both CHO quantity and quality on child size and adiposity.

However, there are two key limitations to the current literature. First, majority of analyses of maternal diet in pregnancy rely on a single assessment during mid or late pregnancy, limiting the capacity to identify potential sensitive windows *in utero* in which diet may have a larger impact on offspring health. Second, most studies evaluate offspring health and body composition during infancy. Little is known regarding how maternal diet during pregnancy correlates with adiposity and metabolic risk in offspring during adolescence. This life stage is a sensitive period for the development of obesity-related disease^(7)^. Furthermore, it is when many metabolic risk factors (e.g. obesity status^(17,18)^, lipid profile^(12)^, blood pressure^(19)^) may be set for life^(19)^.

In this study, we examined trimester-specific associations of maternal CHO intake during pregnancy with adiposity and metabolic health outcomes during peripuberty among participants in the Early Life Exposure in Mexico to Environmental Toxicants (ELEMENT) cohort. We also explored whether these associations were modified by the child’s own CHO intake during adolescence. We hypothesised that higher maternal CHO intake during pregnancy is associated with higher adiposity and metabolic risk during peripuberty, and that this relationship is modified by the child’s own CHO intake.

## Methods

### Study population

Participants are part of the ongoing ELEMENT cohort in Mexico City^(20)^. Pregnant women were recruited in their first trimester of pregnancy in public maternity hospitals between the years 1997 and 2004. For this analysis, our source sample was 250 mother–child pairs who were part of an extramurally funded project to study the effects of prenatal exposures on adiposity and metabolic health across the peripubertal transition (aged 8–14 years). Details on eligibility and enrolment into this sub-study of ELEMENT are published^(9)^. Of the 250 youth recruited, the analytic sample comprised 237 youth who completed an in-person research visit in 2011–2012 (called the adolescent visit for the remainder of this paper) where a trained interviewer administered questionnaires, collected a fasted blood sample and completed an anthropometric evaluation.

### Dietary assessment

We collected dietary intake for mothers during each trimester of pregnancy through use of a semi-quantitative FFQ adapted from the Willet FFQ^(21)^. The FFQ queries usual intake over the last month of 104 foods. Children’s dietary intake over the past week was collected via an interviewer-administered, semi-quantitative FFQ, used in the Mexican Health and Nutrition Survey^(22)^. For children between the ages of 7 and 11 years, the FFQ was administered with the help of the mother^(23)^. For children aged 12 years and older, the FFQ was reported by the child.

Frequency of intake of each food was reported using a range from ‘never’ to ‘6 times a day’. Nutrient content for the foods was verified by two of the following food database sources: Instituto Nacional de Salud Pública 2002, the United States Department of Agriculture, and the Mexican National Institute of Nutrition and Medical Services Salvador Zubiran^(23)^. The kcals for one portion size of the food were multiplied by its frequency of consumption and all foods were summed to estimate usual daily kcal intake. This same procedure was used to estimate other nutrients of interests such as CHO and added sugar. The macronutrients are presented as total energy adjusted using the residuals method^(24)^. This analysis utilised three CHO variables: total CHO, net CHO and total sugars. We calculated the net CHO value as total CHO intake minus fibre which was then energy adjusted. The sugar variable represents total added sugars consumed and therefore differentiates between sugar from natural sources, derived from fruits and vegetables, and added sugars supplied in processed foods.

### Adiposity

At the adolescent visit, trained research staff measured the children’s anthropometry. Weight was measured in kilograms on a digital scale (BAME Model 420; Cátalogo Médico). Height was measured in cm using a calibrated stadiometer (BAME Model 420; Cátalogo Médico). Waist circumference was measured using a non-stretchable measuring tape (QM2000; QuickMedical). The skinfold thicknesses of the sub-scapular and triceps areas were taken in mm using calibrated skin calipers (Lange, Beta Technology). Each of these measures was duplicated, with their averages being used in this analysis. Using weight and height data collected, age- and sex-specific BMI *z*-score values were calculated using the WHO reference^(25)^ as an index of total body size and overall weight status^(26)^. In addition, we assessed waist circumference as a proxy for central visceral adiposity, and the sum (sub-scapular + triceps) and ratio (sub-scapular:triceps) of skinfold thicknesses as markers of overall and subcutaneous adiposity, respectively^(27)^.

### Metabolic risk score

Trained research staff collected blood samples from children after an 8 h fast. We used these samples to measure fasting blood glucose and serum C-peptide. We enzymatically measured fasting blood glucose and quantified C-peptide using an automated chemiluminescence immunoassay (Immulite 1000; Siemens Medical Solutions). We calculated C-peptide insulin resistance (CP-IR) as (fasting serum C-peptide × fasting serum glucose)/405^(28)^. This blood sample also provided blood lipid measurements where total cholesterol (mg/dl), TAG (mg/dl) and HDL (mg/dl) were measured by biochemical analyser (Cobas Mira Plus; Roche Diagnostics) and LDL (mg/dl) was calculated as total cholesterol – HDL – TAG/5^(23)^. We assessed systolic and diastolic blood pressures (mmHg) in the seated position in duplicate, with the average of the two used in the analysis. An internally derived summary risk variable (metabolic syndrome risk *z*-score (MetRisk *z*-score)) was calculated using an average of five-internally standardised *z*-scores for waist circumference, fasting blood glucose, fasting CP-IR (a surrogate for insulin secretion)^(29)^, the ratio of TAG to HDL content and the average of blood pressure measures^(30)^. This summary risk variable has been used in prior ELEMENT studies^(27,30)^ and validated with respect to known metabolic syndrome risk factors in children and with respect to incident type 2 diabetes and CVD in adults^(31)^.

### Covariates

At recruitment into ELEMENT, mothers provided information on current age, reproductive history, lifestyle habits and socio-economic status. During the adolescent visit, a trained paediatrician examined each child and assigned a Tanner stage of 1 (no pubertal development) to 5 (fully developed) based on testicle, breast and pubic hair development^(32–34)^.

#### Data analysis

**Fig. 1:**
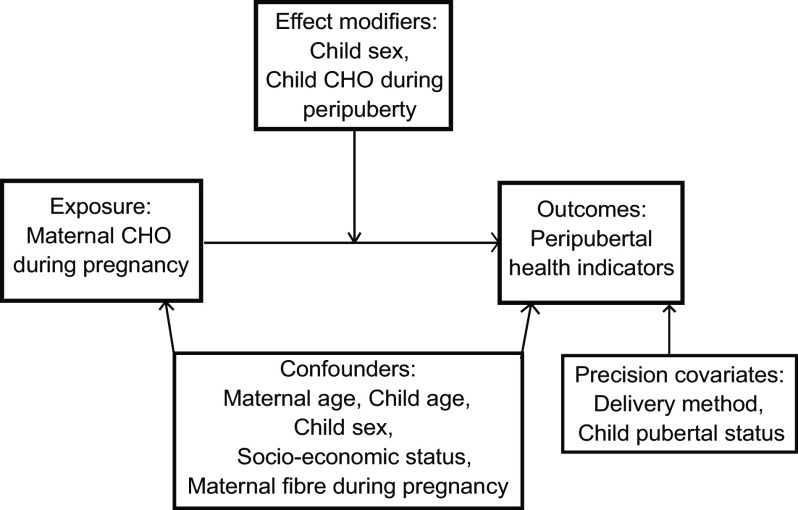
Directed acyclic graph depicting analysis of the current study. The study evaluated maternal intake of CHO during pregnancy (total CHO, net CHO and sugar for trimesters 1, 2 and 3) in quartiles as the exposure in relation to peripubertal BMI *z*-score, waist circumference, sub-scapular and triceps skinfold thickness, ratio of sub-scapular to triceps skinfold thicknesses, fasting glucose, C-peptide, C-peptide insulin resistance (CP-IR), leptin and MetRisk *z*-score as the outcomes. Confounders that were considered were maternal age, child age, child sex, household socio-economic status and maternal fibre intake during pregnancy. Effect modifiers included child sex and child CHO intake during the peripubertal period. Pubertal status of the child during the time of the adolescent visit and delivery method were considered precision covariates with respect to the outcomes only

We first conducted univariate analysis to evaluate distributions of the variables of interest, which include mother’s CHO intake during each trimester of pregnancy. We then carried out bivariate analyses to examine the distribution (mean (sd)) of BMI *z*-score and MetRisk *z*-score across a list of pre-identified variables that we considered as covariates (e.g. confounders, mediators, precision covariates, effect modifiers) in multivariable analysis. Fig. 1 is a directed acyclic graph of key variables of interest.

Next, we examined associations between quartiles of trimester-specific maternal CHO intake (total CHO, net CHO and total sugars) and offspring outcomes during peripuberty using multivariable linear regression models that accounted for maternal age at enrolment, child’s age, and sex, and pubertal status. We assessed maternal CHO intake in quartiles to allow for non-linear associations – an approach that is widely accepted and conventional in nutritional epidemiology (e.g.^(35–37)^). The estimates of interest were pairwise contrasts between the second, third and fourth quartiles of maternal CHO intake *v*. the first quartile as the referent, along with *P*-trend and type 3 *P*-differences to assess statistical significance of associations. We focused on the direction, magnitude and precision of the *ß* estimates and 95 % CI from these analyses.

For all models, we tested for an interaction with sex given differences in metabolism during adolescence. In addition, because the relationship between maternal diet and offspring health could be modified by the child’s own CHO intake, we tested for an interaction between maternal CHO intake during pregnancy and the child’s CHO intake during peripuberty. We did not find any evidence of significant interactions (all *P*-interactions > 0·10); therefore, no stratified analyses were implemented.

In addition to the main analyses, we conducted some sensitivity analyses. First, we evaluated maternal CHO intake using current recommendations for CHO consumption, with 45–65 % of energy representing the acceptable macronutrient distribution range^(38)^ and < 10 % energy from added sugar representing the Institute of Medicine’s recommendation^(27)^. Second, we assessed macronutrient substitution of protein and fat for CHO using multivariate nutrient density models^(39)^. Third, we evaluated including total fibre as a covariate, to determine if glycaemic index was driving these associations. Finally, we explored the impact of adjustment for additional covariates – namely, delivery method and number of household possessions (a measure of socio-economic status) in lieu of maternal education. Additional adjustment for these variables did not materially alter the results, so we did not include them in the final models.

Data were analysed using SAS ® software version 9.4.

## Results

Energy-adjusted CHO intake in pregnancy is shown in Table 1; values are shown as total CHO, net CHO and sugar intake for each trimester. Net CHO intake and total CHO demonstrate similar ranges. Total sugar intake in all three trimesters shows the greatest variance, with a range of 10·7–108·4 g/d between the 5th and 95th percentile.

**Table 1:** Distribution of total energy-adjusted carbohydrate intake (g/d) during pregnancy among 237 ELEMENT mothers

	*n*	Mean	sd	Percentile					
				5th	25th	50th	75th	95th	Max
First trimester									
Total carbohydrate intake (g/d)	228	266·9	33·0	212·2	245·3	266·1	288·5	322·4	363·2
Net carbohydrate intake (g/d)	228	243·6	31·0	194·4	223·2	243·1	265·9	293·9	343·0
Sugar intake (g/d)	228	34·2	16·4	12·3	22·2	31·6	44·6	62·2	89·2
Second trimester									
Total carbohydrate intake (g/d)	235	269·8	33·5	212·2	248·7	268·3	293·4	323·5	379·1
Net carbohydrate intake (g/d)	235	246·9	31·6	194·0	226·0	246·0	267·9	299·9	351·9
Sugar intake (g/d)	235	36·4	17·8	11·9	23·3	33·8	46·2	71·4	89·3
Third trimester									
Total carbohydrate intake (g/d)	236	269·7	35·1	213·7	244·6	269·6	293·8	333·4	366·7
Net carbohydrate intake (g/d)	236	247·4	33·1	196·9	223·5	245·6	269·7	305·9	337·0
Sugar intake (g/d)	236	37·3	18·6	10·7	25·1	34·1	46·4	71·9	108·4

Table 2 displays the associations between maternal and child participant characteristics and child BMI *z*- and MetRisk *z*-scores. Delivery method was associated with both BMI *z*- and MetRisk *z*-scores, with vaginal birth being associated with lower scores for both. Older age during adolescent visit was associated with lower adiposity and higher metabolic risk. Pubertal status was associated with metabolic risk, with prepubertal adolescents having lower MetRisk *z*-scores (–0·16 *v*. 0·15 and –0·10 *v*. 0·22 for males and females, respectively).

**Table 2:** Distribution of BMI *z*-score and metabolic risk phenotype risk *z*-score (MetRisk *z*-score) across characteristics of 237 ELEMENT mother–child pairs

	*n*	BMI *z*-score			*n*	MetRisk *z*-score		
		Mean	sd	*P* †		Mean	sd	*P* †
Overall	237	0·87	1·24		235	0·00	0·63	
Maternal characteristics								
Age at enrolment				0·99				0·89
15–24 years	87	0·95	1·33		87	0·05	0·59	
25–34 years	117	0·75	1·16		117	−0·08	0·60	
35–44 years	32	1·10	1·25		32	0·16	0·75	
Marital status				0·73				0·24
Married	168	0·85	1·22		166	−0·3	0·65	
Single	69	0·92	1·27		69	0·07	0·56	
Maternal education				0·93				0·80
< 10 years	85	0·87	1·24		84	−0·01	0·60	
10–12 years	116	0·88	1·27		115	0·002	0·64	
≥ 13 years	34	0·89	1·15		34	0·03	0·64	
Parity				0·23				0·32
0	84	0·92	1·26		83	0·03	0·60	
1–2	139	0·91	1·19		138	0·01	0·62	
≥ 3	14	0·21	1·38		14	−0·22	0·78	
Smoking during pregnancy				0·97				0·17
Yes	3	0·85	0·62		3	−0·50	0·14	
No	234	0·87	1·24		232	0·01	0·63	
Delivery method				0·06				0·10
C-section	101	1·05	1·26		101	0·08	0·64	
Vaginal	134	0·75	1·21		132	−0·06	0·61	
Delivery weight				0·23				0·40
< 3100 g	111	0·97	1·19		109	0·04	0·59	
> 3100 g	125	0·78	1·27		125	−0·03	0·66	
Child characteristics								
Sex				0·86				0·99
Male	112	0·89	1·19		111	0·00	0·65	
Female	125	0·86	1·28		124	0·00	0·61	
Child’s age				0·11				< 0·0001**
< 10 years	124	0·99	1·21		123	−0·15	0·64	
10–12 years	63	0·78	1·25		62	0·09	0·57	
> 12 years	50	0·69	1·27		50	0·26	0·57	
Carbohydrate intake				0·57				0·96
Q1 (lowest)	59	1·09	1·40		59	0·02	0·64	
Q2	59	0·65	1·30		59	−0·07	0·59	
Q3	59	0·89	1·25		57	0·06	0·66	
Q4 (highest)	59	0·87	0·94		59	−0·01	0·62	
Physical activity (h/week)				0·58				0·17
Q1 (< 17)	33	0·88	1·27		33	0·15	0·66	
Q2 (17–19)	70	0·93	1·36		70	−0·08	0·64	
Q3 (19–21)	59	0·91	1·20		57	0·03	0·61	
Q4 (> 21)	75	0·78	1·14		75	−0·02	0·60	
Time spent watching TV (h/week)				0·68				0·17
Q1 (< 20)	55	0·93	1·04		53	−0·14	0·52	
Q2 (20–25)	57	0·92	1·25		57	0·05	0·70	
Q3 (25–28)	59	0·76	1·27		59	0·02	0·66	
Q4 (> 28)	66	0·88	1·36		66	0·05	0·60	
Pubertal status: males†				0·92				0·01*
Prepubertal	56	0·92	1·28		55	−0·16	0·59	
Pubertal	52	0·90	1·11		52	0·15	0·68	
Pubertal status: females‡				0·65				0·01*
Prepubertal	85	0·82	1·31		84	−0·10	0·64	
Pubertal	40	0·93	1·24		40	0·22	0·46	

* *P* < 0·01,

** *P* < 0·0001

† Represents a test for linear trend where an ordinal indicator is entered into the model as continuous variable, with the exception of binary variables (Wald test).

‡ Puberty was defined as Tanner stage 3–5 (*v*. 1–2) for breast (girls), testicular (boys) and pubic hair (both) development.

Table 3 shows associations of maternal intake of CHO with offspring adiposity. In general, we observed no consistent associations of maternal diet during any trimester with indicators of adiposity in offspring, with non-significant *P*-trends and *P*-differences and all CI for pairwise associations across quartiles containing the null.

**Table 3:** Associations between trimester-specific maternal carbohydrate intake and offspring indicators of adiposity during peripuberty

	*β* (95 % CI) in offspring adiposity†							
Quartile of intake (median g/d)	BMI *z*-score		WC		SS + TR		SS:TR	
	*β*	95 % CI	*β*	95 % CI	*β*	95 % CI	*β*	95 % CI
First trimester								
Total carbohydrate								
Q1 (228·2)	0·00 (Reference)		0·00 (Reference)		0·00 (Reference)		0·00 (Reference)	
Q2 (258·7)	0·29	–0·14, 0·73	3·33	–0·21, 6·87	3·83	–0·25, 7·92	0·06	–0·02, 0·14
Q3 (275·8)	0·36	–0·07, 0·80	2·62	–0·90, 6·14	2·63	–1·43, 6·69	0·003	–0·08, 0·08
Q4 (306·9)	−0·12	–0·55, 0·31	−0·25	–3·76, 3·27	−0·25	–4·30, 3·81	−0·03	–0·12, 0·05
* P*-difference‡	0·10		0·13		0·16		0·18	
* P*-trend	0·82		0·85		0·76		0·16	
Net carbohydrate								
Q1 (206·3)	0·00 (Reference)		0·00 (Reference)		0·00 (Reference)		0·00 (Reference)	
Q2 (233·2)	0·32	–0·12, 0·76	3·58	0·03, 7·14	4·01	–0·09, 8·11	0·07	–0·01, 0·15
Q3 (251·6)	0·17	–0·27, 0·60	1·22	–2·31, 4·75	1·14	–2·93, 5·20	−0·01	–0·09, 0·07
Q4 (282·0)	−0·11	–0·55, 0·32	0·09	–3·44, 3·61	−0·40	–4·46, 3·66	−0·04	–0·12, 0·04
* P*-difference‡	0·26		0·20		0·17		0·07	
* P*-trend	0·46		0·66		0·45		0·07	
Sugar								
Q1 (16·3)	0·00 (Reference)		0·00 (Reference)		0·00 (Reference)		0·00 (Reference)	
Q2 (26·5)	−0·18	–0·62, 0·26	−1·08	–4·65, 2·49	−1·94	–6·04, 2·17	−0·01	–0·09, 0·08
Q3 (36·0)	−0·18	–0·62, 0·26	−1·31	–4·90, 2·28	0·50	–3·64, 4·63	−0·02	–0·10, 0·07
Q4 (54·8)	0·004	–0·45, 0·44	−1·03	–4·65, 2·58	−1·00	–5·16, 3·16	−0·04	–0·12, 0·05
* P*-difference‡	0·76		0·81		0·61		0·68	
* P*-trend	0·99		0·47		0·77		0·22	
Second trimester								
Total carbohydrate								
Q1 (232·9)	0·00 (Reference)		0·00 (Reference)		0·00 (Reference)		0·00 (Reference)	
Q2 (258·7)	−0·25	–0·69, 0·19	−1·81	–5·37, 1·75	−2·74	–6·88, 1·40	−0·05	–0·13, 0·04
Q3 (279·7)	0·04	–0·40, 0·49	1·50	–2·09, 5·10	−1·26	–5·45, 2·92	0·03	–0·05, 0·12
Q4 (308·7)	−0·33	–0·78, 0·12	−2·73	–6·37, 0·90	−2·23	–6·46, 2·00	−0·03	–0·11, 0·06
* P*-difference‡	0·33		0·11		0·68		0·28	
* P*-trend	0·45		0·53		0·52		0·85	
Net carbohydrate								
Q1 (213·7)	0·00 (Reference)		0·00 (Reference)		0·00 (Reference)		0·00 (Reference)	
Q2 (235·7)	0·07	–0·52, 0·37	−0·55	–4·15, 3·05	−0·59	–4·75, 3·58	0·004	–0·08, 0·09
Q3 (254·5)	−0·05	–0·50, 0·40	0·77	–2·86, 4·40	−1·67	–5·87, 2·52	0·05	–0·04, 0·13
Q4 (284·4)	−0·19	–0·65, 0·27	−2·07	–5·77, 1·64	−1·26	–5·55, 3·03	0·01	–0·07, 0·10
* P*-difference‡	0·93		0·51		0·92		0·60	
* P*-trend	0·50		0·55		0·57		0·42	
Sugar								
Q1 (16·7)	0·00 (Reference)		0·00 (Reference)		0·00 (Reference)		0·00 (Reference)	
Q2 (29·1)	−0·01	–0·46, 0·44	−0·03	–3·67, 3·61	−0·47	–4·67, 3·74	0·02	–0·06, 0·11
Q3 (39·8)	−0·17	–0·62, 0·28	−1·35	–5·00, 2·30	−0·81	–5·03, 3·41	−0·03	–0·11, 0·06
Q4 (58·6)	−0·03	–0·50, 0·44	−1·64	–5·43, 2·15	−0·61	–4·99, 3·77	−0·04	–0·12, 0·05
* P*-difference‡	0·90		0·80		0·99		0·55	
* P*-trend	0·87		0·39		0·86		0·35	
Third trimester								
Total carbohydrate								
Q1 (230·2)	0·00 (Reference)		0·00 (Reference)		0·00 (Reference)		0·00 (Reference)	
Q2 (255·7)	0·11	–0·34, 0·56	1·79	–1·87, 5·45	2·37	–1·84, 6·58	0·09	0·01, 0·17
Q3 (280·6)	−0·16	–0·59, 0·28	0·45	–3·12, 4·02	−0·003	–4·11, 4·10	0·05	–0·03, 0·13
Q4 (311·7)	−0·41	–0·85, 0·04	−1·82	–5·46, 1·82	−2·33	–6·52, 1·86	−0·02	–0·10, 0·06
* P*-difference‡	0·14		0·29		0·21		0·05	
* P*-trend	0·046*		0·24		0·18		0·58	
Net carbohydrate								
Q1 (212·6)	0·00 (Reference)		0·00 (Reference)		0·00 (Reference)		0·00 (Reference)	
Q2 (235·4)	0·09	–0·36, 0·53	1·60	–2·02, 5·23	1·19	–2·99, 5·37	0·06	–0·03, 0·14
Q3 (258·4)	−0·02	–0·46, 0·41	1·64	–1·87, 5·15	1·52	–2·52, 5·57	0·04	–0·04, 0·12
Q4 (288·5)	−0·37	–0·81, 0·06	−1·40	–4·91, 2·12	−2·24	–6·29, 1·81	−0·02	–0·10, 0·07
* P*-difference‡	0·22		0·32		0·3		0·32	
* P*-trend	0·11		0·50		0·37		0·79	
Sugar								
Q1 (18·4)	0·00 (Reference)		0·00 (Reference)		0·00 (Reference)		0·00 (Reference)	
Q2 (30·4)	−0·57	–1·01, –0·13	−3·97	–7·55, –0·39	−4·27	–8·40, –0·15	−0·10	–0·18, –0·01
Q3 (38·8)	−0·44	–0·88, 0·01	−2·56	–6·17, 1·05	−1·78	–5·94, 2·38	−0·02	–0·10, 0·07
Q4 (57·7)	−0·44	–0·89, 0·01	−3·45	–7·12, 0·22	−4·07	–8·30, 0·16	−0·04	–0·13, 0·04
* P*-difference‡	0·07		0·12		0·13		0·14	
* P*-trend	0·11		0·11		0·15		0·71	

BMI *z*-score is calculated according to the WHO growth reference for children aged 5–19.

WC, waist circumference (cm).

SS + TR: the sum of sub-scapular and triceps skinfolds (mm).

SS:TR: the ratio of sub-scapular to triceps skinfold thickness.

* *P*-difference or *P*-trend <0·05.

† Model is adjusted for maternal age, child sex, child age and pubertal status.

‡ *P*-difference is the result of a Wald *χ* ^2^test.

Table 4 shows associations of maternal CHO intake with offspring metabolic parameters (glycaemia, C-peptide, CP-IR, leptin and MetRisk *z*-score). Our primary finding was that maternal net CHO intake during the first trimester and intake of total CHO during the third trimester were each associated with offspring C-peptide and CP-IR in an inverse J-shaped manner, wherein the highest quartile of intake corresponded with lowest values of the biomarkers. During the first trimester, the second, third and fourth quartiles of maternal total CHO intake were associated with 0·26 (95 % CI –0·15, 0·68), 0·20 (95 % CI –0·21, 0·61) and –0·31 (95 % CI –0·72, 0·11) ng/ml C-peptide in offspring, respectively, with a significant *P*-difference across the quartiles (*P*-differences = 0·05), despite the fact that the CI for pairwise estimates contained the null and thus were not statistically significant. Similarly, the second, third and fourth quartiles of maternal net CHO intake during the first trimester corresponded with 0·46 (95 % CI 0·05, 0·88), 0·08 (95 % CI –0·33, 0·49) and –0·29 (95 % CI –0·70, 0·12), respectively (*P*-difference = 0·01). We noted the same pattern of association between maternal net and total CHO intake during the first trimester in relation to offspring CP-IR, as well as with respect to maternal net and total CHO intake during the third trimester in relation to offspring C-peptide, CP-IR and leptin (Table 4).

**Table 4:** Associations between trimester-specific maternal carbohydrate intake and offspring metabolic biomarkers during peripuberty

	*β* (95 % CI) in offspring measures of metabolic health†									
Quartile of intake (median g/d)	Fasting glucose (mg/dl)		C-peptide (ng/ml)		CP-IR		Leptin (ng/ml)		MetRisk *z*-score	
	*β*	95 % CI	*β*	95 % CI	*β*	95 % CI	*β*	95 % CI	*β*	95 % CI
First trimester										
Total carbohydrate										
Q1 (228·2)	0·00 (Reference)		0·00 (Reference)		0·00 (Reference)		0·00 (Reference)		0·00 (Reference)	
Q2 (258·7)	1·40	–1·95, 4·75	0·26	–0·15, 0·68	0·07	–0·05, 0·19	1·14	–1·90, 4·19	0·17	–0·05, 0·38
Q3 (275·8)	2·58	–0·73 (5·90	0·20	–0·21, 0·61	0·07	–0·05, 0·18	2·06	–0·95, 5·08	0·15	–0·06, 0·36
Q4 (306·9)	−0·30	–3·63, 3·03	−0·31	–0·72, 0·11	−0·07	–0·19, 0·05	−1·03	–4·06, 1·99	−0·07	–0·28, 0·15
* P*-difference‡	0·38		0·05		0·08		0·13		0·13	
* P*-trend	0·87		0·13		0·22		0·95		0·45	
Net carbohydrate										
Q1 (206·3)	0·00 (Reference)		0·00 (Reference)		0·00 (Reference)		0·00 (Reference)		0·00 (Reference)	
Q2 (233·2)	2·99	–0·34, 6·32	0·46	0·05, 0·88	0·14	0·02, 0·25	1·61	–1·44, 4·65	0·25	0·03, 0·46
Q3 (251·6)	0·24	–3·07, 3·56	0·08	–0·33, 0·49	0·01	–0·10, 0·13	1·24	–1·79, 4·28	0·07	–0·14, 0·29
Q4 (282·0)	−1·08	–4·40, 2·23	−0·29	–0·70, 0·12	−0·07	–0·18, 0·05	−0·91	–3·94, 2·12	−0·05	–0·26, 0·17
* P*-difference‡	0·13		0·01*		0·01*		0·23		0·07	
* P*-trend	0·23		0·049*		0·07		0·72		0·28	
Sugar										
Q1 (16·3)	0·00 (Reference)		0·00 (Reference)		0·00 (Reference)		0·00 (Reference)		0·00 (Reference)	
Q2 (26·5)	4·51	1·21, 7,81	0·31	–0·11, 0·73	0·11	–0·00, 0·23	−1·31	–4·36, 1·75	0·10	–0·11, 0·32
Q3 (36·0)	3·17	–0·17, 6·51	0·14	–0·28, 0·57	0·05	–0·07, 0·16	−0·29	–3·38, 2·79	0·04	–0·18, 0·26
Q4 (54·8)	0·93	–2·41, 4·27	−0·04	–0·46, 0·39	−0·00	–0·12, 0·12	−1·13	–4·22, 1·97	−0·002	–0·22, 0·22
* P*-difference‡	0·06		0·43		0·22		0·90		0·81	
* P*-trend	0·97		0·58		0·58		0·96		0·63	
Second trimester										
Total carbohydrate										
Q1 (232·9)	0·00 (Reference)		0·00 (Reference)		0·00 (Reference)		0·00 (Reference)		0·00 (Reference)	
Q2 (258·7)	2·23	–1·14, 5·59	0·14	–0·29, 0·56	0·07	–0·05, 0·19	−2·12	–5·19, 0·95	−0·05	–0·27, 0·17
Q3 (279·7)	0·28	–3·10, 3·67	0·12	–0·31, 0·55	0·03	–0·09, 0·15	0·31	–2·77, 3·39	0·04	–0·18, 0·26
Q4 (308·7)	3·74	0·31, 7·16	−0·26	–0·69, 0·17	−0·04	–0·16, 0·08	−2·66	–5·78, 0·46	0·04	–0·18, 0·27
* P*-difference‡	0·14		0·23		0·33		0·18		0·84	
* P*-trend	0·12		0·34		0·52		0·37		0·50	
Net carbohydrate										
Q1 (213·7)	0·00 (Reference)		0·00 (Reference)		0·00 (Reference)		0·00 (Reference)		0·00 (Reference)	
Q2 (235·7)	3·41	0·04, 6·79	0·29	–0·13, 0·72	0·07	–0·05, 0·19	−1·39	–4·48, 1·70	0·05	–0·17, 0·27
Q3 (254·5)	1·93	–1·44, 5·30	0·14	–0·29, 0·56	−0·01	–0·13, 0·11	0·63	–2·45, 3·72	0·08	–0·14, 0·30
Q4 (284·4)	4·38	0·94, 7·82	−0·12	–0·56, 0·31	−0·04	–0·16, 0·09	−2·32	–5·47, 0·84	0·11	–0·11, 0·34
* P*-difference‡	0·09		0·22		0·31		0·27		0·74	
* P*-trend	0·048*		0·58		0·82		0·42		0·27	
Sugar										
Q1 (16·7)	0·00 (Reference)		0·00 (Reference)		0·00 (Reference)		0·00 (Reference)		0·00 (Reference)	
Q2 (29·1)	−0·63	–4·04, 2·79	0·33	–0·09, 0·76	0·11	–0·01, 0·23	−0·36	–3·48, 2·76	0·05	–0·17, 0·27
Q3 (39·8)	1·62	–1·83, 5·06	−0·05	–0·48, 0·38	0·04	–0·08, 0·16	−1·16	–4·30, 1·99	−0·06	–0·28, 0·17
Q4 (58·6)	1·34	–2·22, 4·89	−0·16	–0·60, 0·29	−0·00	–0·12, 0·12	−0·79	–4·04, 2·45	0·04	–0·19, 0·27
* P*-difference‡	0·56		0·11		0·17		0·95		0·80	
* P*-trend	0·32		0·29		0·38		0·67		0·97	
Third trimester										
Total carbohydrate										
Q1 (230·2)	0·00 (Reference)		0·00 (Reference)		0·00 (Reference)		0·00 (Reference)		0·00 (Reference)	
Q2 (255·7)	0·43	–3·03, 3·88	0·18	–0·24, 0·61	0·05	–0·07, 0·16	1·83	–1·24, 4·91	0·15	–0·07, 0·37
Q3 (280·6)	−0·35	–3·73, 3·04	−0·15	–0·57, 0·27	−0·05	–0·16, 0·07	2·11	–0·90, 5·13	0·05	–0·17, 0·26
Q4 (311·7)	0·76	–2·70, 4·21	−0·42	–0·85, 0·01	−0·10	–0·22, 0·02	−2·52	–5·60, 0·56	−0·13	–0·35, 0·09
* P*-difference‡	0·94		0·04*		0·10		0·01*		0·12	
* P*-trend	0·84		0·02*		0·04*		0·15		0·18	
Net carbohydrate										
Q1 (212·6)	0·00 (Reference)		0·00 (Reference)		0·00 (Reference)		0·00 (Reference)		0·00 (Reference)	
Q2 (235·4)	−3·77	–7·16, –0·38	−0·27	–0·69, 0·16	−0·10	–0·22, 0·02	1·51	–1·57, 4·58	0·01	–0·21, 0·23
Q3 (258·4)	−0·58	–3·88, 2·71	−0·38	–0·79, 0·03	−0·11	–0·23, 0·00	2·24	–0·75, 5·23	0·02	–0·19, 0·23
Q4 (288·5)	−0·93	–4·23, 2·37	−0·54	–0·96, –0·13	−0·15	–0·26, –0·03	−1·63	–4·62, 1·36	−0·12	–0·33, 0·10
* P*-difference‡	0·15		0·05		0·06		0·08		0·58	
* P*-trend	0·86		0·01*		0·01*		0·39		0·31	
Sugar										
Q1 (18·4)	0·00 (Reference)		0·00 (Reference)		0·00 (Reference)		0·00 (Reference)		0·00 (Reference)	
Q2 (30·4)	−2·54	–5·93, 0·86	−0·48	–0·90, –0·05	−0·14	–0·26, –0·02	−3·10	–6·18, –0·02	−0·18	–0·40, 0·04
Q3 (38·8)	−0·17	–3·58, 3·23	−0·21	–0·64, 0·21	−0·07	–0·19, 0·05	−1·38	–4·47, 1·71	−0·09	–0·31, 0·12
Q4 (57·7)	−0·89	–4·38, 2·59	−0·41	–0·84, 0·03	−0·12	–0·24, 0·00	−1·58	–4·74, 1·58	−0·17	–0·40, 0·05
* P*-difference‡	0·43		0·10		0·07		0·23		0·31	
* P*-trend	0·90		0·13		0·11		0·47		0·19	

CP-IR, C-peptide associated insulin resistance score.

MetRisk *z*-score: a cumulative *z*-score calculated by taking the average of five internally standardised *z*-scores for waist circumference, blood glucose, C-peptide, TAG/(HDL) and (systolic+diastolic blood pressure)/2.

* *P*-difference or *P*-trend<0·05.

† Model is adjusted for maternal age, child sex, child age and pubertal status.

‡ *P*-difference is the result of a Wald *χ* ^2^ test.

### Sensitivity analyses

#### Use of acceptable macronutrient distribution range and Institute of Medicine sugar recommendations

Table 5 shows associations of maternal total CHO intake during each trimester according to the acceptable macronutrient distribution range for CHO intake, categorised as ‘below’, ‘at’ and ‘above’ the acceptable macronutrient distribution range. Overall, these thresholds were not associated with any of the offspring outcomes.

**Table 5:** Associations of maternal intake in relation to nutritional recommendations during pregnancy and offspring adiposity and a metabolic syndrome risk *z*-score (MetRisk *z*-score) in peripuberty

	*β* (95 % CI)*			
Recommendations (% energy intake)	BMI *z*-score†		MetRisk *z*-score‡	
	*β*	95 % CI	*β*	95 % CI
Sugar recommendations				
First trimester				
Sugar < 10 % energy	0·00 (reference)		0·00 (reference)	
Sugar > 10 % energy	−0·12	–0·49, 0·25	−0·03	–0·22, 0·15
* P*-difference§	0·52		0·74	
Second trimester				
Sugar < 10 % energy	0·00 (reference)		0·00 (reference)	
Sugar > 10 % energy	−0·16	–0·54, 0·21	−0·07	–0·26, 0·11
* P*-difference§	0·39		0·44	
Third trimester				
Sugar < 10 % energy	0·00 (reference)		0·00 (reference)	
Sugar > 10 % energy	−0·09	–0·44, 0·27	−0·07	–0·24, 0·10
* P*-difference§	0·63		0·42	
AMDR recommendations				
First trimester				
< 45 % energy	−0·06	–0·47, 0·36	0·03	–0·18, 0·23
45–65 % energy	0·00 (reference)		0·00 (reference)	
> 65 % energy	0·06	–0·38, 0·39	−0·05	–0·24, 0·14
* P*-difference§	0·73		0·45	
Second trimester				
< 45 % energy	−0·28	–0·68, 0·12	−0·08	–0·28, 0·12
45–65 % energy	0·00 (reference)		0·00 (reference)	
> 65 % energy	−0·42	–0·79, –0·05	−0·19	–0·38, –0·01
* P*-difference§	0·44		0·25	
Third trimester				
< 45 % energy	−0·41	–0·80, –0·01	−0·14	–0·33, 0·06
45–65 % energy	0·00 (reference)		0·00 (reference)	
> 65 % energy	−0·18	–0·55, 0·18	−0·12	–0·30, 0·06
* P*-difference§	0·33		0·94	

AMDR, acceptable macronutrient distribution range.

* Model is adjusted for maternal carbohydrate intake, child sex, child age and pubertal status.

† BMI *z*-score is calculated according to the WHO growth reference for children aged 5–19 years.

‡ MetRisk *z*-score: a cumulative *z*-score calculated by taking the average of five internally standardised *z*-scores for waist circumference, blood glucose, C-peptide, TAG/(HDL) and (systolic plus diastolic blood pressure) divided by 2.

§ *P*-difference is the result of a Wald *χ* ^2^ test.

### Macronutrient substitutions

Nutrient substitution analyses did not indicate that our findings were due to differences in fat or protein intake. Protein substituting total CHO in the third trimester was the only estimate to show a statistically significant association with reduction in MetRisk *z*-score (data not shown; available upon request).

## Discussion

### Summary

In this study of 237 mother–child pairs in Mexico City, we found that highest (i.e. fourth quartile) maternal intake of total and net CHO during pregnancy, particularly during the first and third trimesters, were associated with C-peptide and CP-IR in an inverse J-shaped manner where the highest quartile of maternal intake corresponded with lowest values of the metabolic biomarkers in offspring at 8–14 years of age. We did not observe any association with respect to offspring adiposity.

### Comparison to existing literature

To date, most studies exploring effects of CHO exposure during gestation and long-term health outcomes in offspring have examined associations of *in utero* exposure to gestational diabetes mellitus (GDM), which results in high circulating glucose levels, with offspring health outcomes. These studies have generally found that a diagnosis of GDM and/or severity of maternal hyperglycaemia correlates with worse offspring metabolic profile^(9)^. For example, a study of 164 mother–child pairs in China demonstrated that children of women with GDM had higher cord blood insulin levels and higher blood pressures and lower HDL at 8 years of age than those whose mothers were normoglycaemic during pregnancy^(40)^. Similarly, in a recently published analysis from the Healthy Start cohort, Francis *et al*. found an association between tertiles of maternal HbA1c (an indicator of maternal glucose levels in the prior 3 months) during mid-pregnancy was associated with higher fasting glucose in offspring at age 4–7 years^(41)^.

Some studies have explored effects of maternal CHO intake during pregnancy in relation to offspring body size at birth, though the results have been inconsistent. For example, in the Healthy Start study in Colorado, Crume *et al*.^(42)^ found that greater maternal intake of total CHO and sugar during pregnancy was associated with lower fat mass in offspring at birth^(42)^. Similarly, McKenzie and colleagues^(43)^ found that offspring of women in the fourth quartile of CHO intake had lower body weight and body fatness at birth than those in the second quartile, although this association failed to reach statistical significance^(43)^. On the other hand, in a UK-based study of 1196 mother–infant pairs, Sharma *et al*. found that each additional 10 g of CHO intake per day during pregnancy was associated with a 4 g increase in offspring weight at birth^(44)^. Conversely, in an older study of 1082 mother–infant dyads in New Jersey, Scholl *et al*. reported that mothers who consumed a low dietary glycaemic index were more likely to deliver children that were small for gestational age^(16)^. Finally, in a recent analysis, Chen and colleagues reported that higher CHO intake during pregnancy was associated with higher offspring BMI in early childhood^(14)^. Discrepancies in findings from the above studies could be due to different methods of assessing CHO intake (e.g. nutrient or energetic density methods by Crume *et al*. and McKenzie *et al*. *v*. glycaemic index in Scholl *et al*.), differences in background characteristics in study populations (including age of participants), differential adjustment for covariates in multivariable models and/or residual confounding.

There are few possible explanations for our unexpected finding of an inverse J-shaped trend between maternal CHO intake during pregnancy and offspring C-peptide and CP-IR, wherein youth born to women with the highest CHO intake had the lowest values for these biomarkers. The first is residual confounding by maternal conditions during the perinatal period. For example, maternal pre-pregnancy overweight or obesity^(45)^ and excess gestational weight gain^(46)^ during pregnancy are determinants of offspring adiposity and metabolic risk^(9)^ that we do not have available for ELEMENT participants. The second is possible reverse causation by a diagnosis of GDM, which is typically made during the second trimester. Specifically, women with GDM are typically placed on a strict low- CHO diet and increased physical activity to reduce circulating glucose levels. Presumably, women with GDM would consume fewer CHO following diagnosis, whereas normoglycaemic women, who are often underweight or of healthy weight prior to pregnancy^(47,48)^, are likely to be represented in the upper quartiles of CHO intake. In this scenario, the effect of high maternal CHO intake in the latter part of pregnancy could be capturing the effect of a healthy and/or lower pre-pregnancy BMI, which are consistently related to more favourable offspring health outcomes^(45,49,50)^. However, this does not explain the similar associations that we observed with respect to maternal CHO intake during the first trimester. Another possibility is that the adverse consequences of *in utero* CHO exposure may not manifest until older ages. Animal models of Western diet, which is both high in simple CHO and saturated and trans fats, in pregnancy demonstrate delayed onset of obesity and related comorbidities. In a rat model, administration of a high fat and high-simple-sugar diet during pregnancy produced progeny that developed increased body weight and higher blood leptin levels than those whose mothers had been fed a normal control diet^(51)^. These effects were only present in the offspring after reaching adulthood^(51)^. A third possible explanation may be that endocrine disrupting chemicals, like phthalates found in food packaging^(52)^ and bisphenol A often found in beverage packaging^(53)^, may drive the association with child metabolic measurements. Finally, given the largely null findings, we acknowledge the possibility that we may have been insufficiently powered to detect associations in this current study with the available sample size. Future studies with a large sample size and more complete covariate data on maternal perinatal conditions are required to confirm our findings.

### Strengths and limitations

The current study has several strengths. Use of trimester-specific associations of diet with offspring adiposity and metabolic risk allows for more detailed understanding of critical periods within gestation. Inclusion of both subcutaneous and central measures of adiposity allows for more sensitivity and may facilitate inference of compartmentalisation of fat tissue during development. The use of multiple measures of metabolic health provided a detailed picture of adolescent metabolic health in relation to maternal prenatal diet.

Findings must also be interpreted in light of limitations. First, as mentioned earlier, we do not have information on maternal pre-pregnancy BMI, gestational weight gain or gestational glucose tolerance – all of which are likely associated with maternal CHO intake as well as offspring health outcomes. Second, because we relied on FFQ to ascertain dietary intake, we cannot rule out the possibility of recall bias, although energy adjusting the CHO intake variables does improve precision of our estimates. Third, because pubertal stage occurs on a continuous spectrum, categorising this phenomenon as an ordinal variable at the time of the peripubertal research visit likely does not fully capture inter-individual variability in tempo of maturation. Thus, our results may still be vulnerable to residual confounding by pubertal status. Fourth, given the relatively small sample size, it is possible that we were underpowered to detect associations between maternal diet and subclinical biomarkers of metabolic risk in youth. Finally, ELEMENT is comprised of low- to middle-income participants in urban Mexico; these findings may not be generalisable to other populations of different ethnic composition, socio-economic status and geographic location.

## Conclusions

In this study, we found that women who consumed the highest amounts of CHO during the first and third trimesters of their pregnancies had children with lower C-peptide and CP-IR but no other metabolic biomarker or adiposity indicators during peripuberty. The results of this analysis are valuable for understanding peripubertal health of a very specific population, adolescents in Mexico City. Additional research is needed to confirm these findings in other populations and to elucidate underlying mechanisms.
